# The Suboptimal QLV Ratio May Indicate the Need for a Left Bundle Branch Area Pacing-Optimized Cardiac Resynchronization Therapy Upgrade

**DOI:** 10.3390/jcm13195742

**Published:** 2024-09-26

**Authors:** Péter Ezer, Kitti Szűcs, Réka Lukács, Tamás Bisztray, Gábor Vilmányi, István Szokodi, András Komócsi, Attila Kónyi

**Affiliations:** 1 Heart Institute, Medical School, University of Pécs, 7624 Pécs, Hungary; szucs.kitti@pte.hu (K.S.); lukacs.reka@pte.hu (R.L.); gabor.vilmanyi@pte.hu (G.V.); istvan.szokodi@pte.hu (I.S.); komocsi.andras@pte.hu (A.K.); konyi.attila@pte.hu (A.K.); 2 Szentágothai Research Center, University of Pécs, 7622 Pécs, Hungary; 3 Department of Informatics, University of Oslo, 0316 Oslo, Norway; tamasbi@ifi.uio.no

**Keywords:** cardiac resynchronization therapy, left bundle branch area pacing, left ventricular lead implantation, QLV ratio, LOT-CRT, CRT response

## Abstract

**Background**: The QLV ratio (QLV/baseline QRS width) is an established intraoperative-measurable parameter during cardiac resynchronization therapy (CRT) device implantation, potentially predicting the efficacy of electrical resynchronization. **Methods**: Left bundle branch area pacing-optimized CRT (LOT-CRT) is a novel approach with the potential to improve both responder rate and responder level in the CRT candidate patient group, even when an optimal electro-anatomical left ventricular lead position is not achievable. In our observational study, 72 CRT-defibrillator candidate patients with a QRS duration of 160 ± 12 ms were consecutively implanted. Using a QLV-ratio-based implant strategy, 40 patients received a biventricular CRT device (Biv-CRT) with an optimal QLV ratio (≥70%). Twenty-eight patients with a suboptimal QLV ratio (<70%) were upgraded intraoperatively to a LOT-CRT system. Patients were followed for 12 months. **Results**: The postoperative results showed a significantly greater reduction in QRS width in the LOT-CRT patient group compared to the Biv-CRT patients (40.4 ± 14 ms vs. 32 ± 13 ms; *p* = 0.024). At 12 months, the LOT-CRT group also demonstrated a significantly greater improvement in left ventricular ejection fraction (14.9 ± 8% vs. 10.3 ± 7.4%; *p* = 0.001), and New York Heart Association functional class (1.2 ± 0.5 vs. 0.8 ± 0.4; *p* = 0.031), and a significant decrease in NT-pro-BNP levels (1863± 380 pg/mL vs. 1238 ± 412 pg/mL; *p* = 0.012). Notably, the LOT-CRT patients showed results comparable to Biv-CRT patients with a super-optimal QLV ratio (>80%) in terms of QRS width reduction and LVEF improvement. **Conclusions**: Our single-center study demonstrated the feasibility of a QLV-ratio-based implantation strategy during CRT implantation. Patients with a LOT-CRT system showed significant improvements, whereas Biv-CRT patients with a super-optimal QLV ratio may not be expected to benefit from an additional LOT-CRT upgrade.

## 1. Introduction

Cardiac resynchronization therapy (CRT) reduces the risk of heart-failure-related hospitalizations, decreases all-cause mortality, and improves the quality of life and functional status in symptomatic heart failure patients with reduced left ventricular ejection fraction (HFrEF) in candidates who have left bundle branch block (LBBB), or non-specific intraventricular conduction delay (ns-IVCD) with a QRS duration over 150 ms [1,2,3,4].

CRT with a coronary sinus (CS) lead means vigorously recommended therapeutic modality in the upper patient population according to the current available European Society of Cardiology (ESC) resynchronization guidelines, even despite great advancement in cardiac conduction system pacing (CSP) therapy modalities [1,5].

Conventional CRT is delivered by implanting a lead through the coronary sinus to capture the latest activating left ventricular epicardial area, aiming to correct the inter/intraventricular conduction delay. However, this approach is associated with significant drawbacks, including a relatively high rate of procedural failure, high left ventricular pacing thresholds, lead instability, dislodgement, phrenic nerve stimulation, and potential suboptimal electro-anatomical position of the lead. Moreover, with conventional biventricular pacing systems, many patients fail to derive significant benefits from resynchronization [6]. Epicardial left ventricular lead implantation via surgical thoracotomy was mentioned as a safe and proven alternative for selected patients with transvenous implantation failure [7].

Currently, there are limited methods to predict the effectiveness of CRT intraoperatively. Coronary sinus side branch lead positioning is a major and important factor for CRT response; thus, the left ventricular (LV) lead should preferably be placed in a region with considerable electric delay. Measurements of the electro-anatomical position of the LV lead may be used to predict the efficacy of CRT.

QLV is defined as the interval from the onset of the QRS from the surface ECG to the left-ventricular-sensed R wave of the left ventricular lead electrogram. The QLV ratio is obtained by normalizing this interval with the baseline QRS width. The QLV and QLV ratio (QLV/baseline QRS width) measurements are simple, non-time-consuming, and reliable methods [8,9,10]. Based on previous studies, if the QLV ratio is below 70%, it indicates a suboptimal electro-anatomical position of the LV lead and lowers the therapeutic effectiveness of CRT therapy, manifesting in non-favorable clinical outcomes such as heart failure hospitalization or death compared to CRT-implanted patients with an optimal (≥70%) QLV ratio [11,12].

Recently, perioperative CRT optimization strategies gained importance; thus, suboptimal atrioventricular (AV) timing may be the most prevalent modifiable factor influencing clinical response [13]. LV-only or fusion pacing appears to be effective in patients with intact AV conduction and LBBB. The effect might be augmented by left ventricular multi-point-pacing (MPP) [14].

Conduction system pacing (CSP) and prominently left bundle branch area pacing (LBBAP) have recently emerged as a potential alternative to traditional biventricular CRT (Biv-CRT) therapy. By using LBBAP, the patient’s native conduction system or the left ventricular subendocardium can be stimulated, achieving an improved ventricular activation pattern by bypassing the block in the conduction system. Patients with successful LBBAP for CRT show favorable outcomes compared to conventional CRT patients in terms of reduced left ventricular activation time (LVAT) and paced QRS width. Further on, they exhibit a more homogeneous left ventricular electro-mechanical activation pattern, potentially improving outcomes regarding left ventricular volumetric remodeling parameters, as well as functional and heart failure status, compared to conventional CRT optimization strategies [15,16,17,18,19].

Even though a significant proportion of patients fail to achieve QRS correction from an LBBAP-only strategy and need advanced resynchronization strategies for distal conduction disease, LBBAP pacing strategies along with implanted coronary sinus LV electrodes, known as the left bundle branch area pacing-optimized CRT (LOT-CRT) system, may provide meaningful clinical benefits, although currently we lack randomized controlled data in this field [20,21].

The criteria for selecting patients for LOT-CRT are not yet clearly defined. However, observational studies have demonstrated the feasibility and efficacy of using LOT-CRT in patients with heart failure and advanced intraventricular conduction delay (IVCD) [22,23].

According to the recent ESC pacing guidelines, CS lead implantation is mandatory to achieve proper resynchronization in symptomatic HFrEF patients with LBBB/non-specific-IVCD QRS morphology and wide (>150 ms) QRS duration [1].

The concept of completing and optimizing the CRT system by implanting additional conduction system pacing (CSP) or left bundle branch area pacing (LBBAP) lead based on the measured QLV ratio is an emerging approach among LOT-CRT implantation indications. A successful LBBAP-optimized CRT technique may benefit patients with suboptimal electrical LV lead position in terms of multiple electro-mechanical and clinical improvements.

We aimed to test the feasibility of a QLV-ratio-based resynchronization strategy for patients with IVCD (LBBB or ns-IVCD) patients and to determine if LOT-CRT shows superior performance compared to conventional Biv-CRT in terms of clinical response to resynchronization therapy. For patients with a suboptimal electrical delay at the definitive CS lead position, an additional LBBAP pacing lead was added intraoperatively to complete the LOT-CRT system.

## 2. Materials and Methods

### 2.1. Study Population

In a single-center observational study, consecutive patients with heart failure with reduced ejection fraction (HFrEF) and IVCD (LBBB or ns-IVCD) were recruited. The inclusion criteria were as follows:

1. Chronic heart failure symptoms were classified as New York Heart Association (NYHA) class II to IV/a.
2. A baseline left ventricular (LV) ejection fraction ≤ 40%.
3. Participants had to be on stable doses of heart failure therapies of guideline-recommended medical therapy for HFrEF (ACE-inhibitors, neprilysin inhibitors, SGLT-2 inhibitors, mineralocorticoid receptor antagonist (MRA) and beta receptor blockers).
4. Sinus rhythm or permanent atrial fibrillation with LBBB or ns-IVCD and a QRS duration > 150 ms.
5. Patients with permanent AF underwent AV node ablation to maximize biventricular pacing percentage if a high average resting ventricular rate (>75/min) limiting appropriate biventricular pacing ratio was present.

Patients were assigned to LOT-CRT or BiV-CRT depending on the feasibility of postero-lateral/lateral CS LV electrode implantation and the intraoperative QLV ratio measurement. This study enrolled consecutive patients who met the above inclusion criteria at the Heart Institute, University of Pécs Medical School (Pécs, Hungary), from January 2022 to December 2023.

The study had the following exclusion criteria:

1. Participants had to be between 18 and 90 years of age.
2. Patients with right bundle branch block (RBBB) QRS morphology were excluded.
3. Patients with prior pacing- or defibrillator device or any cardiac implantable device were not included.
4. Participants with a diagnosis of therapy-refractory end-stage heart failure, or other malignant diseases that potentially limit life expectancy to less than 1 year, were excluded.
5. Participants had to be willing to return for required follow-up visits; non-cooperating individuals were excluded from the follow-up schedule.
6. Pregnant individuals were excluded from the study.

### 2.2. Ethics and GDPR Compliance

Data collection was performed following international regulations regarding the protection of personal information and data. All subjects gave their informed consent for inclusion in the study before participation. The study was conducted in accordance with the Declaration of Helsinki, and the protocol was approved by the Regional Research Ethics Committee of the University of Pécs Medical School (Pécs, Hungary) as an investigator-initiated observational study on 12 August 2020. Record number: PTE-6600/2020.

### 2.3. Device Implantation Procedure, CS Side Branch Selection, and QLV Measurement

Seventy-two consecutive patients were implanted with CRT defibrillator systems with initial DF-1/IS-1 shock leads (Biotronik Intica Neo HF-T™ Biotronik, Berlin, Germany) DF-1/IS-1 shock lead connector, IS-4 LV electrode connector). All implantation procedures were performed by two experienced operators in this field. Procedures were in consonance with ESC European Heart Rhythm Association device implantation recommendations [24]. Cephalic vein cut-down was applied as standard procedure for inserting right ventricular DF1-/IS-1 shock electrode. Fluoroscopy-guided axillary venous punctures were performed three times for every patient to acquire venous access for lead implantation.

CS cannulation and selective CS venography was applied for selecting potential postero-lateral/lateral side branches for lead implantation. LV lead implantation attempts were made following the results of the MADIT-CRT trial; left ventricular postero-lateral/lateral CS side branches, and only basal- or midventricular positions, were considered to be appropriate lead positions [25]. No attempt was made to use posterior, or anterior, CS side branches. All implantations utilized quadripolar (IS-4) left ventricular lead (Biotronik Sentus ProMRI QP™ Biotronik, Berlin, Germany). LV electrode implantation was unsuccessful in three patients due to inappropriate anatomical properties of the CS side branch and, in one case, because of persistent phrenic nerve stimulation of LV lead. A bail-out LBBAP procedure was performed in these patients.

Intraoperative measurements of QLV and QLV ratio were taken from all potential LV lead pacing sites (LV1-LV4). With the help of the quadripolar lead, the pace-sense analyzer (PSA) and recording system were used to collect QLV measurements from all potential LV pacing/sensing sites. PSA connection was made sequentially for every LV pacing/sensing pole (LV1-LV2/LV2-LV3/LV3-LV4). The PSA ventricular sensing threshold was standardized and set to 2.5 mV. QLV value for each site was defined as the interval between the earliest onset 12 lead surface QRS to the center of the largest peak, whether negative or positive, of the LV bipolar intracardiac electrogram during a cardiac cycle.

The intraoperative decision on electrode placement was based on the LV pole with the longest QLV value and highest QLV ratio measurement in addition to acceptable pacing threshold values (<3.0 V and 1.0 ms) without phrenic nerve stimulation.

RV shock electrode was placed in the right ventricular apex. Sensed and paced inter-electrode distance (IED) was measured between the RV tip-ring and selected LV pole.

Based on QLV ratio measurements, three categories were defined: (1) QLV ratio below 70% was considered suboptimal. (2) QLV ratio of equal or above 70% was considered optimal for electro-anatomical lead properties. (3) Among optimal LV leads, QLV ratio higher than 80% was considered super-optimal lead positions.

**Figure 1:**
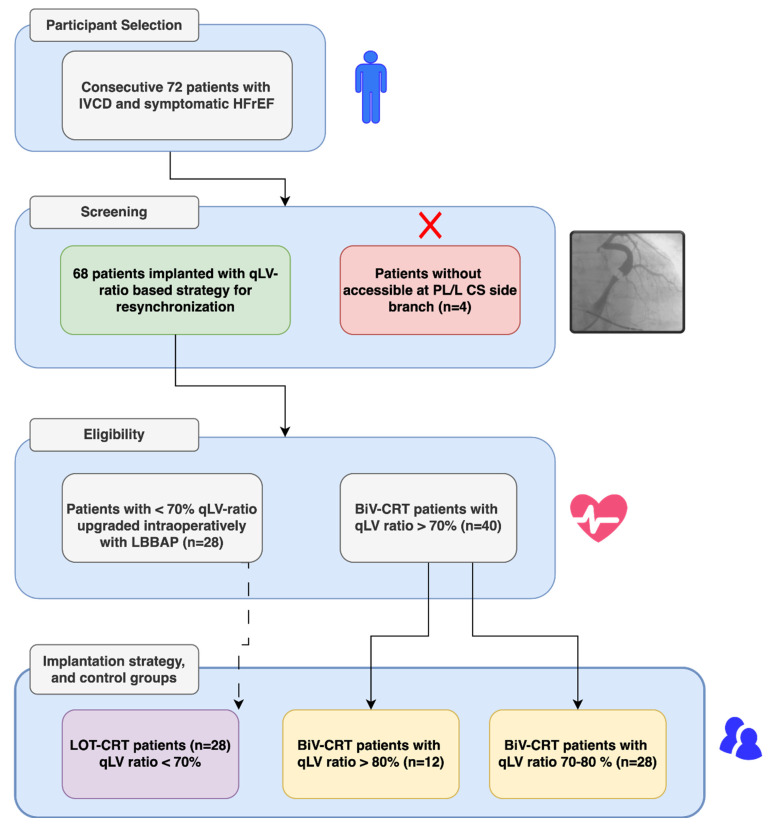
Flowchart depicting inclusion of patients and the resynchronization device implantation according to the QLV-ratio-based strategy. Abbreviations: Biv-CRT: biventricular pacing cardiac resynchronization therapy; CS: coronary sinus; PL: postero-lateral; L: lateral; qLV ratio: QLV value divided by baseline QRS width in milliseconds; LV: left ventricular; LBBAP: left bundle branch area pacing; LOT-CRT: left bundle branch area pacing-optimized cardiac resynchronization therapy; IVCD: intraventricular conduction delay; HFrEF: heart-failure-reduced left ventricular ejection fraction.

If a suboptimal LV lead position (QLV ratio: <70%) was identified, an additional LBBAP lead was implanted, which was the case for 28 out of 68 patients. For these implantations, Biotronik Selectra 3D™ (Biotronik, Berlin, Germany) preformed conduction system pacing sheaths and Biotronik Solia S 60™ (Biotronik, Berlin, Germany) leads were used for stylet stylet-driven LBBAP procedure. Before LBBAP lead implantation, pace mapping of the right ventricular septum was performed; thus, optimal lead implantation location was identified. A maximum of 2 attempts were made to achieve selective/non-selective left bundle branch (LBB) capture. If no LBB capture was possible, then left ventricular septal capture (LVS) was accepted as the final pacing result. Definition of LBB and/or LVS capture was based on (1) appearance of RBBB morphology QRS or R prime paced QRS complex in lead V1, (2) consequent decrease in paced QRS width, (3) stimulation to R wave peak time in lead V6, and (4) V1-V6 R wave interpeak interval. Successful LBB/LVS capture was confirmed by output-dependent paced QRS morphology change. Pacing capture results were accepted as criteria for LBBAP according to the European Heart Rhythm Association (EHRA) consensus document on conduction system pacing [26]. Next, the IS-1 pin of the DF-1/IS-1 shock lead was capped, and the LBBAP IS-1 lead was connected to the CRT generator’s RV IS-1 pace/sense port. Patient selection and implantation strategy is summarized in Figure 1.

### 2.4. Patient Follow-Up

Twelve-lead surface ECG was performed, and patients were optimized in terms of AV- and ventriculo-ventircular (VV) timing on postoperative day 1. The shortest AV delay was obtained by decreasing the AV delay from 200 ms with −10 ms steps, and then the VV delay was sequentially programmed. AV and VV pacing configurations with the shortest paced QRS complex on 12-lead surface ECG were considered for best available timing configuration.

Patients were scheduled for in-clinic visits 3 months later with complete device interrogation, and surface ECG tests were performed to prove CSP/LV capture in LOT-CRT and Biv-CRT patients.

All the patients were followed with an automated daily data-transmission-based remote monitoring system (Biotronik Home Monitoring™, Biotronik GmBH, Berlin, Germany), which has been proved as a safe and effective method in patient surveillance [27].

Automatic threshold tests were switched OFF; automatic daily lead impedance and sensing tests and general remote monitoring alerts were programmed ON. Remote interrogation of the device was performed 3 times monthly, and in-patient follow-up visits with repeated transthoracic echocardiography and NT-pro-BNP measurements were performed at one-year follow-up visits.

### 2.5. Study Endpoints

The primary objective of this study was to compare 12-month clinical outcomes of patients in the Biv-CRT group with electro-anatomically acceptable LV electrodes (defined as an intraoperative measurement of QLV ratio ≥ 70%) with patients with suboptimal (<70% QLV ratio) LV electrodes who also received an additional LBBAP lead (LOT-CRT system).

Patients in the Biv-CRT group with super-optimal QLV ratio (>80%) and LV lead position (Biv-CRT SOL) were evaluated as a separate subgroup for patient outcomes.

Periprocedural parameters including QRS width reduction and decrement in R wave peak time measurement in ECG lead V6 (V6 RWPT), as well as procedural and fluoroscopic times, were analyzed.

At the end of the 12-month follow-up, a control visit was performed to measure the left ventricular ejection fraction by transthoracic echocardiography, to monitor NT-pro-BNP kinetics, and to reassess functional status.

Three independent expert echocardiographic readers were blinded for pre-operative and follow-up assessments.

### 2.6. Statistical Analysis

Categorical variables were expressed as counts and percentages, and continuous variables were reported as means ± SD. Missing data were not replaced; all available data were used for sample distribution evaluation. Normality was assessed using the Kolmogorov–Smirnov test.

Paired or independent comparisons were performed using a Student *t*-test if the data were normally distributed, while the Wilcoxon signed-rank test was used for nonparametric data. For categorical variables, the Fisher exact test was used.

An ordinal logistic regression was conducted to assess the effect of the treatment group (LOT-CRT versus Biv-CRT) on changes in NYHA classification. The dependent variable was the post-treatment NYHA classification, coded as an ordinal variable with levels I, II, III, and IV. The independent variable was the treatment group. The model was fitted using maximum likelihood estimation. Pearson’s correlation test and linear regression analysis were performed for statistical correlation testing and linear regression analysis.

It should be noted that no sample size calculations were performed before study completion. A *p*-value of <0.05 was considered significant.

All statistical analyses and graphical material presentation were performed in GraphPad Prism version 10.2.3 and IBM SPSS statistics version 28.0.1.

## 3. Results

### 3.1. Baseline Characteristics

Of the 72 patients initially screened, 68 patients had accessible postero-lateral or lateral coronary sinus side branches, and a further 4 were excluded due to a lack of suitable left ventricular pacing position. In these four cases, bail-out LBBAP was performed, thus gaining a left bundle branch pacing-optimized implantable defibrillator (LOT-ICD) system. These particular patients were not followed according to the baseline study design.

Primary Biv-CRT defibrillator implantation was attempted in all patients according to the current ESC guidelines of pacing therapy in HFrEF and significant (>150 ms) IVCD of non-RBBB QRS morphology.

The baseline patient characteristics, medications, and echocardiographic parameters are shown in Table 1.

Briefly; age, gender, NT pro-BNP levels, comorbidities, basic echocardiographic parameters (such as LVEF, LV end-systolic, and end-diastolic diameters), and medication history were not significantly different between the Biv-CRT and LOT-CRT groups. Of the 68 patients, 40 (58.8%) had an optimal-positioned LV lead with a QLV ratio equal to or above 70%, making them an appropriate candidate for Biv-CRT therapy. The remaining 28 patients (41.2%) had suboptimal CS LV electrode positions in terms of QLV ratio and were candidates for LOT-CRT implantation with an intraoperative LBBAP attempt. Notably, the Biv-CRT patients had a significantly higher likelihood of LBBB QRS morphology than the LOT-CRT patients (81% vs. 64%; *p* = 0.032) at baseline, and, among the LOT-CRT patients, significantly more ns-IVCD patients were present than in the Biv-CRT group (34% vs. 20%; *p* = 0.041)

### 3.2. Evaluation of Procedural Outcomes

**Figure 2:**
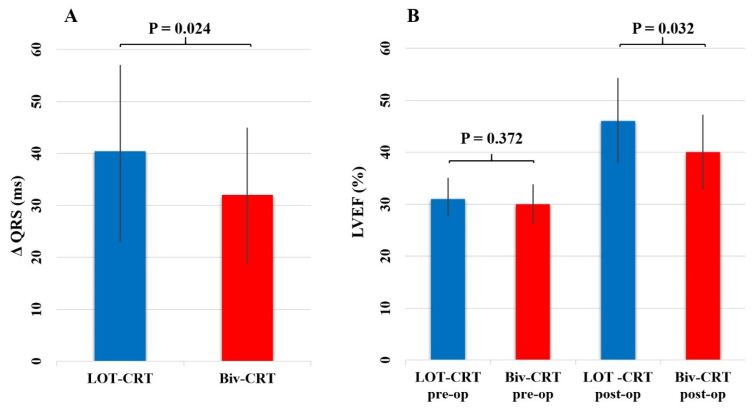
Changes in QRS duration and left ventricular ejection fraction (LVEF) in LOT-CRT and Biv-CRT patient groups. (**A**): Change in QRS duration (ΔQRS). This panel illustrates the difference in QRS duration reduction between the LOT-CRT and Biv-CRT patient groups. The LOT-CRT group demonstrated a significantly greater reduction in QRS duration compared to the Biv-CRT group (*p* = 0.024). (**B**): Left ventricular ejection fraction (LVEF) preoperative and postoperative. This panel shows the preoperative and postoperative LVEF percentages for the LOT-CRT and Biv-CRT patient groups. Postoperatively, the LOT-CRT group exhibited a significantly greater improvement in LVEF compared to the Biv-CRT group (*p* = 0.032). Preoperative LVEF levels were not significantly different between the groups (*p* = 0.372). Error bars represent standard deviations.

The success rate for this implantation strategy was 94.4%. The mean procedure duration for BiV-CRT implantation was 87 ± 56 min, while the average duration for LOT-CRT device implantation was 119.5 ± 59.5 min (*p* = 0.032). Mean fluoroscopy time was also significantly longer at LOT-CRT implantations (28± 15 min) compared to Biv-CRT implantations (21 ± 8 min; *p* = 0.045). In terms of paced inter-electrode distance (Paced IED) (138 ± 14.6 ms vs. 152 ± 21 ms; *p* = 0.024) and QLV ratio (55.27 ± 12% vs. 83.22 ± 11.1%; *p* = 0.018), the LOT-CRT patients had significantly worse outcomes than the Biv-CRT patients. Despite a non-optimal CS electrode position, the reduction in QRS duration (40.4 ± 17 ms vs. 32 ± 13 ms; *p* = 0.024) (Figure 2A) and shorter V6 R wave peak time (V6 RWPT; 71.4 ± 13.7 ms vs. 98.76 ± 12.6 ms; *p* = 0.0018) were significantly superior in the LOT-CRT patient group versus the Biv-CRT patients (Table 2). Interestingly, four patients without accessible CS side branches were scheduled for a “bail-out” LBBAP procedure, thus gaining a LOT-ICD system. These patients had a mean procedural QRS width reduction of 35 ± 7 ms and reached a mean 81 ± 9.2 ms in V6 RWPT, in which the values are comparable to the LOT-CRT and Biv-CRT patient outcomes; although, considering the patient group size of the LOT-ICD patients, statistical relevance is constrained.

**Figure 3:**
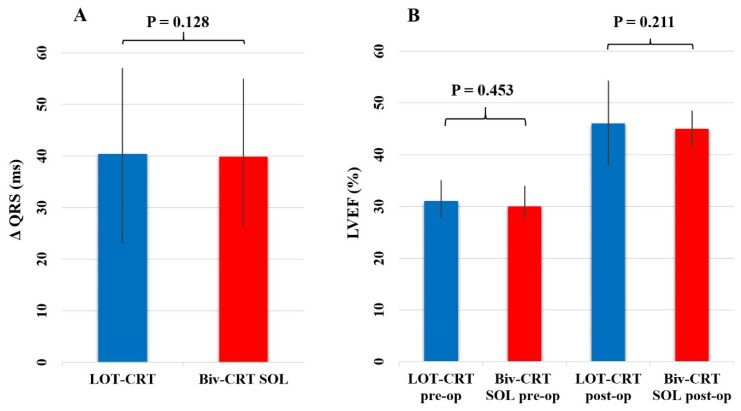
Comparison of QRS duration reduction and left ventricular ejection fraction (LVEF) improvement in LOT-CRT and Biv-CRT SOL patients. (**A**): Change in QRS duration (ΔQRS). This panel displays the difference in QRS duration reduction between the LOT-CRT and Biv-CRT super-optimal lead (SOL) patient groups. There was no significant difference in QRS duration reduction between the two groups (*p* = 0.128). (**B**): Left ventricular ejection fraction fraction (LVEF) preoperative and postoperative. This panel shows the preoperative and postoperative LVEF percentages for the LOT-CRT and Biv-CRT SOL patient groups. Postoperatively, there was no significant difference in LVEF improvement between the LOT-CRT and Biv-CRT SOL groups (*p* = 0.21). Preoperative LVEF levels were also not significantly different between the groups (*p* = 0.45). Error bars represent standard deviations.

Twelve patients with left ventricular lead implantation achieved a qLV ratio greater than 80% and were categorized as super-optimal lead (SOL) Biv-CRT patients. The QRS duration reduction (−40.4 ± 19 ms vs. −38.9 ± 18 ms; *p* = 0.128) was not significantly different in the LOT-CRT patients compared to the Biv-CRT SOL patients (Figure 3A).

### 3.3. Follow-Up Results

**Figure 4:**
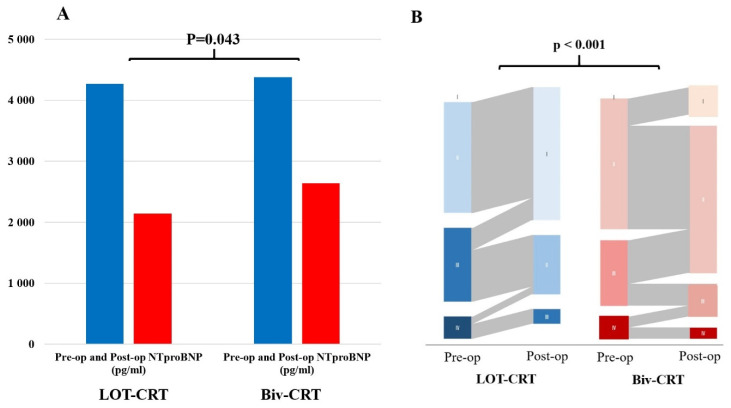
Changes in NT-pro-BNP levels and NYHA functional class. (**A**): Change in NT-pro-BNP levels. This panel illustrates the changes in NT-pro-BNP levels (pg/mL) in the LOT-CRT and conventional Biv-CRT patient groups. The LOT-CRT group showed a significantly greater reduction in NT-pro-BNP levels compared to the Biv-CRT group (*p* = 0.043). (**B**): Sankey diagram demonstrates NYHA functional class preoperative and postoperative. This panel shows the distribution of NYHA functional class in the LOT-CRT and Biv-CRT patient groups, both preoperatively and postoperatively. Postoperatively, the LOT-CRT group exhibited a significantly greater improvement in the NYHA functional class compared to the Biv-CRT group (*p* < 0.001); furthermore, the likelihood of functional improvement was significantly higher in the LOT-CRT patient group (*p* < 0.001).

In LOT-CRT patients, a significant improvement in LVEF was observed (14.9 ± 9.3% vs. 10.3 ± 7.4%; *p* = 0.001) (Figure 2B), and there was a significant decrease in NT-pro-BNP levels (1863 ± 380 pg/mL vs. 1238 ± 412 pg/mL; *p* = 0.012, respectively) (Figure 4A).

The ordinal logistic regression model demonstrated a significant effect of the treatment group on the change in NYHA classification. The coefficient for the LOT-CRT group was 2.2542 (*p* < 0.001), indicating a higher likelihood of improvement in NYHA classification compared to the Biv-CRT group (Figure 4B), whereas the NYHA functional class improvement (1.2 ± 0.5 vs. 0.8 ± 0.4; *p* = 0.031) was significantly better compared to the Biv-CRT patients. Control LVEF (46.3 ± 7.8% vs. 45.2 ± 3.5%; *p* = 0.211) did not differ significantly between the LOT-CRT and Biv-CRT SOL patient group (Figure 3B).

### 3.4. Analysis of on Intraoperative Electrical Measurements

An ordinal Pearson’s correlation test and logistic regression analysis were conducted to assess potential intraoperative measurable parameters in predicting postoperative LVEF improvement (ΔLVEF).

**Figure 5:**
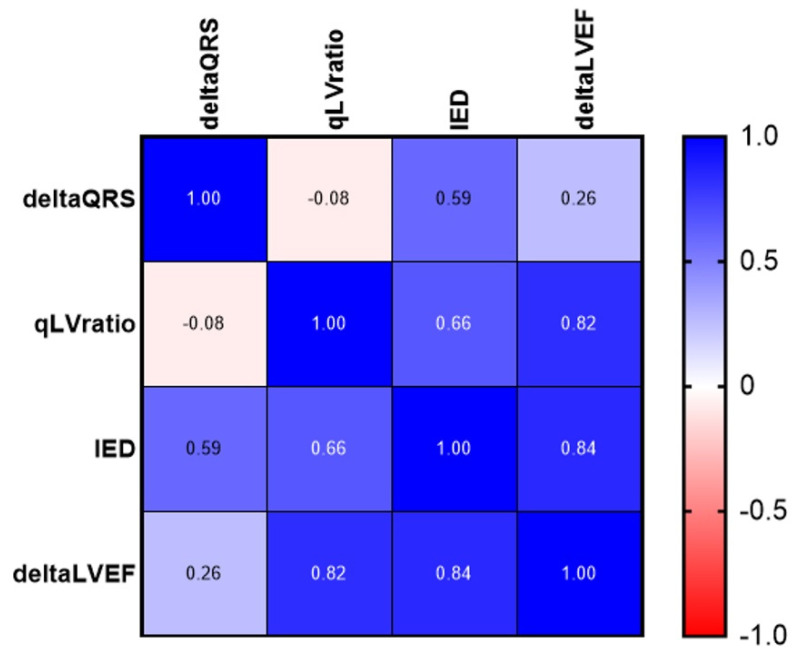
Correlation heatmap matrix representing left ventricular ejection fraction (LVEF) improvement and different intraoperative measurements. This figure shows a heatmap displaying the correlation coefficients (Rho) between different variables: the change in left ventricular ejection fraction (ΔLVEF; deltaLVEF) and various intraoperative measurements: change in QRS duration (ΔQRS; deltaQRS), QLV ratio, and paced inter-electrode distance (IED). The color scale on the right represents the strength and direction of the correlations, with blue indicating positive correlations and red indicating negative correlations.

Pearson’s correlation matrix revealed a strong positive correlation between intraoperative QLV ratio measurements and ΔLVEF, with a correlation coefficient of 0.82. Additionally, there was a strong positive correlation between paced IED and ΔLVEF, with a correlation coefficient of 0.84. However, the reduction in procedural QRS width did not show a significant correlation with ΔLVEF (correlation coefficient; r = 0.26) (Figure 5).

**Figure 6:**
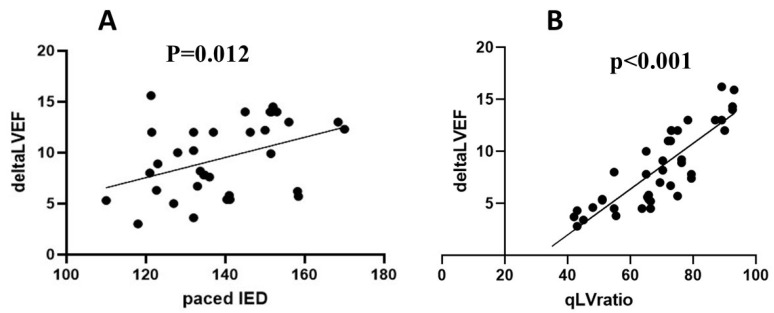
Scatter plots of correlation analysis of intraoperative measurements and postoperative left ventricular ejection fraction (LVEF) improvement (ΔLVEF) in Biv-CRT patient group. (**A**): Correlation between paced inter-electrode distance (IED) and ΔLVEF. This panel displays the scatter plot analysis of the correlation between paced inter-electrode distance (IED) and the change in left ventricular ejection fraction (ΔLVEF). The analysis shows a significant positive correlation (*p* = 0.012; r = 0.68). (**B**): Correlation between qLV ratio and ΔLVEF. This panel shows the scatter plot analysis of the correlation between QLV ratio and the change in left ventricular ejection fraction (ΔLVEF). The analysis indicates a highly significant positive correlation (*p* < 0.001; r = 0.88). Each point represents an individual patient’s data. The lines represent the best-fit regression lines.

Scatter plot analysis in the Biv-CRT patient group demonstrated a positive correlation between paced IED and ΔLVEF (*p* = 0.012). As the paced IED increases, ΔLVEF tends to increase as well (Figure 6A). Furthermore, a strong positive correlation was observed in the QLV ratio and ΔLVEF with a *p*-value of less than 0.01, indicating a highly statistically significant relationship (Figure 6B).

## 4. Discussion

In our study, we observed 72 HFrEF patients with IVCD who were implanted with CRT devices using an intraoperative QLV-ratio-based implantation strategy. The main goal was to determine if patients with a suboptimal QLV ratio (<70%) could benefit from an additional LBBAP lead (LOT-CRT) in order to improve outcomes and match those of patients with an optimal QLV ratio (≥70%) receiving conventional CRT.

Our findings demonstrate that LOT-CRT is feasible and may lead to better clinical outcomes for patients with suboptimal QLV ratios during CRT implantation, bringing their outcomes closer to those of the subgroup with super-optimal (>80% QLV) ratios.

The main findings over a 12-month follow-up are as follows: (1) The QLV-ratio-based strategy was feasible in a high number of patients; however, there was a significant difference in procedural time, with the LOT-CRT group requiring an average of 30 min longer than conventional CRT implantations. (2) Despite suboptimal CS lead positioning, the LOT-CRT group experienced significantly greater reductions in QRS duration and left ventricular activation time (LVAT), as measured by V6 R wave peak time (RWPT). (3) The LOT-CRT group showed significantly greater improvements in LVEF, as well as more substantial reductions in NT-pro-BNP levels and NYHA functional class after 12 months of follow-up. (4) In the LOT-CRT arm, the reduction in QRS duration reached levels comparable to those observed in the CRT subgroup with super-optimal QLV ratios (>80%). (5) The LVEF of LOT-CRT patients also reached that of the Biv-CRT subgroup with super-optimal QLV ratios.

Several earlier studies have assessed outcomes with QLV-based resynchronization strategies. Accumulating evidence supports that among the possible electrical parameters of the LV activation measurement of QLV and QLV ratio appears to be a promising parameter with a potential to optimize electrical resynchronization during CRT implantation procedure.

Gold et al. identified QLV measured at CRT implantations as a strong predictor of acute hemodynamic response verified by pacing–simultaneous invasive measurements of LV dP/dt. The QLV interval was measured at the LV pacing site. In a multivariate analysis, QLV during biventricular pacing was an independent predictor of hemodynamic response, with a 1.7% increase in LV dP/dt for every 10 milliseconds prolongation of QLV value [8]. In 426 patients undergoing CRT implantation, QLV was found to be strongly and independently associated with reverse remodeling and quality of life measures after CRT implantation [9]. However, a subgroup analysis of the SMART-AV (SmartDelay Determined AV Optimization: A Comparison to Other AV Delay Methods Used in Cardiac Resynchronization Therapy) trial described RV–LV inter-electrode distance measurement to be a more suitable parameter compared to QLV for predicting patient prognosis related to resynchronization. It is important to note, however, that this latter study included a meaningful proportion of patients with complete right bundle branch block, for whom QLV has no additional value in predicting potential resynchronization efficiency during the CRT implantation [10]. Singh et al. investigated 71 consecutive patients undergoing CRT implantation and found that a reduced QLV/baseline QRS value (QLV ratio) of less than 50% was associated with worse clinical outcomes, defined as the combined endpoint of hospitalization for heart failure and/or all-cause mortality at 12 months. The QLV ratio was significantly higher in acute responders (69.6% ± 23.9) in the non-ischemic etiology group [11]. Roubicek et al. found that a QLV ratio greater than 70% was a significant and strong predictor for heart failure hospitalization and mortality in CRT-implanted 329 patients who were followed for an average of 3.3 ± 1.9 years [12].

The ENHANCE-CRT trial enrolled and randomized 284 subjects with non-LBBB QRS morphology in a 2:1 ratio between a QLV-based implantation approach and an anatomical implantation approach. Patients were implanted with a quadripolar LV lead cardiac resynchronization therapy defibrillator system. The observed difference in the composite score based on paced QRS morphology and width did not show statistical significance. The analysis of freedom from heart failure hospitalization and cardiac death showed similar results in the QLV-guided and control arm. Subjects with a QRS duration of <150 ms or >150 ms also resulted in similar outcomes. In addition, there were no significant differences between the two interventional arms either in the quality of life or LVEF. The trial highlighted the analysis by QLV ratio (quartiles revealed no significant difference in CRT response rates between quartiles [28]).

There are some fundamental differences to note between this latter study and our study. The patient selection criteria of the ENHACE-CRT study resulted in the inclusion of 44% of patients with a baseline QRS width between 120 and 149 ms; furthermore, 60% of patients received a CRT-defibrillator device with a baseline RBBB QRS morphology. In this patient group, QLV measurements have limited feasibility as the activation of the left bundle and accompanying myocardium may remain intact, resulting in no electrical delay observed on the LV electrogram. In accordance with this, a recent meta-analysis indicates that patients with right bundle branch block morphology do not benefit in terms of clinical outcomes from conventional biventricular resynchronization therapy [4].

Extensive randomized data of patients, including long-term clinical efficacy, confirm that CRT implantation in an appropriately selected HFrEF population significantly improves patient morbidity and mortality. The highest clinical efficacy is observed in patients with HFrEF, sinus rhythm, wide QRS duration (>150 ms), and LBBB QRS morphology. Procedural QRS width reduction in the LBBB patient population is known to be a strong predictor of survival [2,29,30]. However, a significant proportion of patients do not benefit from conventional CRT implantation, thus increasing the responder rate remains a crucial aspect of resynchronization therapy [6].

The concept of conduction system pacing has opened new possibilities not only in conventional pacemaker treatment but also in the resynchronization therapy of patients with heart failure. Currently, in the absence of large-scale multicenter randomized trials, there is no clear recommendation for resynchronization therapy using LBBAP stimulation, but the data published so far are very encouraging in terms of QRS width reduction and LVEF improvement [16,17,31,32,33,34].

LOT-CRT provides a hybrid paced-fusion depolarization wavefront of LBBAP and LV epicardial pacing, potentially resulting in a more optimal electro-mechanical activation pattern of the ventricles, even in advanced heart failure [19,20].

Jastrzebski and co-investigators followed 91 patients with baseline IVCD or paced right ventricular rhythm implanted with LOT-CRT devices. This international registry demonstrated that LOT-CRT is a feasible and safe method with the potential for superior resynchronization effects. The study found that LOT-CRT provides better electrical resynchronization, QRS width reduction, and clinical improvement compared to Biv-CRT. The findings underscore that LOT-CRT could be a superior alternative, particularly when only suboptimal electrical resynchronization is achieved with Biv-CRT [21].

Similarly, Chen et al. found significantly lower total mortality in patients implanted with LOT-CRT compared to conventional Biv-CRT patients in a non-randomized observational study of 85 patients with advanced HFrEF and IVCD [22].

Despite that these studies show a potential for a superior resynchronization effect in certain subpopulations, large-scale, multicenter, and randomized controlled clinical trials are needed to further evaluate the clinical benefits and safety of LOT-CRT in CRT candidate patients and evaluate potential emerging indications for LOT-CRT device implantation at a suboptimal procedural QLV ratio measurement.

## 5. Limitations

This study should be interpreted considering certain methodological limitations.

First, this is a single-center observational study with a limited sample size. No sample size calculation was performed to evaluate the event rates and incidence of the outcomes in the specific population before study completion. These factors also restrict the statistical power and generalizability of the findings.

The QLV value and QLV ratio were measured at the final lead position, representing the longest QLV interval and highest QLV ratio percentage for each patient. Additionally, the choice of lead position was predominantly on the postero-lateral, lateral wall basal/midventricular region, limiting the ability to fully evaluate the relationship between the QLV and lead position. No anterior or posterior LV lead positions were used in this study.

The observational study design did not allow for us to analyze the total effects of confounding variables, such as comorbidities, medications, or previous interventions. This oversight can result in biased estimates of the treatment effect.

Finally, an echocardiographic measure of left ventricular ejection fraction improvement is not an ideal marker of reverse remodeling per se. A left ventricular end-systolic volume change of at least 15% is a better predictor of survival and clinical outcomes with CRT. However, “harder” endpoints such as survival or hospitalization were not powered endpoints in this study, and the short follow-up period precluded a more complete assessment of heart failure events.

## 6. Conclusions

The QLV-ratio-based resynchronization strategy was found to be feasible and effective in supporting decision-making during surgery. In this observational study, LOT-CRT showed that patients with non-right bundle branch block (RBBB) QRS morphology and suboptimal QLV ratio at implantation had narrower QRS durations and better echocardiographic and clinical responses compared to those who received biventricular CRT.

This study showed that LOT-CRT had a better outcome versus conventional Biv-CRT with a QLV ratio between 70% and 80% as well. Noteworthy, LOT-CRT could be a choice with a QLV ratio below 80%. A QLV ratio interval between 70% and 80% may be considered as a “grey zone” for an LBBAP upgrade; individualized decision making and a thorough weighing of cost–benefit for the patient should be proposed in this situation.

Definitive results in QRS width reduction and LVEF improvement achieved by LOT-CRT were comparable to those of Biv-CRT in patients with a super-optimal LV lead position (>80% QLV ratio). These patients might not see the further benefit of adding LBBAP lead to a Biv-CRT system.

**Figure 7:**
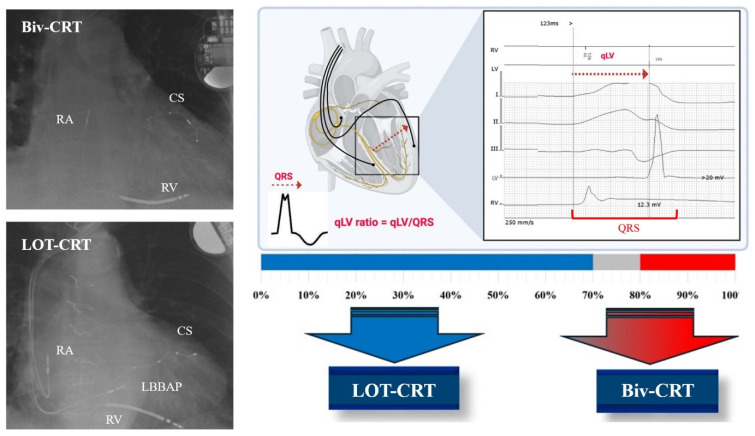
QLV-ratio-based resynchronization strategy for cardiac resynchronization therapy. After LV lead placement, QLV and QLV ratio are assessed. If the QLV value (ms)/baseline QRS width (ms) is below 70%, an intraoperative upgrade with LBBAP lead (IS-1 connector) is performed, thus resulting in a LOT-CRT system. If the QLV ratio is above 80%, then no additional benefit was seen compared to the LOT-CRT system. If the QLV ratio falls between the 70 and 80% “grey-zone”, then a potential upgrade with LBBAP is a decision based on clinical situation, cost–benefit weighing, and preference of the operating physician.

In conclusion, LOT-CRT might provide a promising alternative for HFrEF patients with IVCD and suboptimal CS anatomy or QLV ratio for conventional Biv-CRT. Further randomized controlled trials will be essential in this field to determine the exact recommendations. Figure 7 summarizes a QLV-ratio-based resynchronization strategy proposed based on our results.

## Figures and Tables

**Table 1:** Baseline patient characteristics.

Characteristic	LOT-CRT (*n* = 28)	Biv-CRT (*n* = 40)	*p*-Value
Age, years	69 ± 7	70 ± 8.1	NS
Sex: male/female	18/10	26/14	NS
LBBB, *n* %	18, (64)	32 (80)	*p* < 0.05
Ns-IVCD, *n* %	10, (34)	8 (20)	*p* < 0.05
NT pro-BNP level, pg/mL	4269 ± 4165	4380 ± 4978	NS
Scar burden on cMR, %	12 ± 10	9.6 ± 6	NS
Medical history			
HTN, *n* (%)	22, (78.5)	31, (77.5)	NS
DM, *n* (%)	11, (39.2)	16 (40)	NS
Permanent AF, *n* (%)	7, (25)	16 (40)	NS
Previous myocardial infarction, *n* (%)	10, (36)	14, (35)	NS
COPD, *n* (%)	2, (7.1)	3, (7.5)	NS
NYHA class III-IVa, *n* (%)	8, (28.5)	11, (27.5)	NS
Mean baseline NYHA class	2.67 ± 0.6	2.58 ± 0.8	NS
Baseline echocardiographic parameters			
LVEF, %	31.4 ± 3.7	30 ± 3.8	NS
LVEDD, mm	55.7± 5.6	58.8 ± 9.3	NS
LVESD, mm	45 ± 6.8	44 ± 6	NS
Baseline medications			
ACEi or ARB, %	16, (57.1)	23, (57.5)	NS
ARNI, %	12, (42.8)	17, (42.5)	NS
SGLT-2 inhib., %	16, (57.1)	23, (57.5)	NS
Beta blocker, %	22, (78.5)	31, (77.5)	NS
MRA, %	24, (85.7)	34, (42.5)	NS

Abbreviations: AF; atrial fibrillation; ACEi: angiotensin-converting enzyme inhibitor; ARB: angiotensin receptor blocker; ARNI: angiotensin receptor neprilysin inhibitor; cMR: cardiac magnetic resonance imaging; COPD: chronic obstructive pulmonary disease; DM: diabetes mellitus type 2; HTN: hypertension; LVEF: left ventricular ejection fraction; LVEDD: left ventricular end-diastolic diameter; LVESD: left ventriuclar end-systolic diameter; LBBB: left bundle branch block; SGLT-2 inhibitor: sodium-glucose co-transporter 2 inhibitor; MRA: mineralocorticoid receptor antagonist; Ns-IVCD: non-specific intraventricular conduction delay; NYHA: New York Heart Association; NS: non-significant (*p* > 0.05).

**Table 2:** Procedural outcomes.

Characteristic	LOT-CRT (*n* = 28)	Biv-CRT (*n* = 40)	*p*-Value
QLV, ms	89 ± 12.1	134 ± 12	*p* < 0.01
QLV ratio, %	55.27 ± 12	83.22 ± 11.1	*p* < 0.05
Paced IED, ms	138 ± 14.6	152 ± 21	*p* < 0.05
QRS preprocedural, ms	161 ± 15	160 ± 11	NS
QRS after, ms	119 ± 14	134 ± 16	*p* < 0.01
QRS reduction, ms	40.4 ± 17	32 ± 13	*p* < 0.05
LBB capture, *n* (%)	20 (72%)	n.a.	n.a.
LVS capture, *n*	8 (18%)	n.a.	n.a.
V6 RWPT, ms	71.4 ± 13.74	98.76 ± 12.6	*p* < 0.01
V1-V6 RWPi, ms	47.42 ± 11.3 ms	n.a.	n.a.
Mean procedural time (min.)	119.5 ± 59.6	87 ± 56.5	*p* < 0.05
Mean fluoroscopy time (min.)	28 ± 15	21 ± 8	*p* < 0.05

Abbreviations: QLV: left ventricular activation marker on intracardiac channel marked on surface ECG; qLV ratio: QLV divided by the baseline QRS width; IED: inter-electrode distance; LBB: left bundle branch; LVS: left ventricular septal; RWPT: R wave peak time; RWPi: R wave interpeak interval; NS: non-significant (*p* > 0.05); n.a.: not available.

## Data Availability

The data presented in this study are available on request from the corresponding author. The data are not publicly available due to Hungarian legal regulations.

## References

[1] Glikson M., Nielsen J.C., Kronborg M.B., Michowitz Y., Auricchio A., Barbash I.M., Barrabés J.A., Boriani G., Braunschweig F., Brignole M. (2021). 2021 ESC Guidelines on cardiac pacing and cardiac resynchronization therapy: Developed by the Task Force on cardiac pacing and cardiac resynchronization therapy of the European Society of Cardiology (ESC) With the special contribution of the European Heart Rhythm Association (EHRA). Eur. Heart J..

[2] Sipahi I., Chou J.C., Hyden M., Rowland D.Y., Simon D.I., Fang J.C. (2012). Effect of QRS morphology on clinical event reduction with cardiac resynchronization therapy: Meta-analysis of randomized controlled trials. Am. Heart J..

[3] Auricchio A., Lumens J., Prinzen F.W. (2014). Does Cardiac Resynchronization Therapy Benefit Patients with Right Bundle Branch Block. Circ. Arrhythm. Electrophysiol..

[4] Friedman D.J., Al-Khatib S.M., Dalgaard F., Fudim M., Abraham W.T., Cleland J.G.F., Curtis A.B., Gold M.R., Kutyifa V., Linde C. (2023). Cardiac Resynchronization Therapy Improves Outcomes in Patients with Intraventricular Conduction Delay But Not Right Bundle Branch Block: A Patient-Level Meta-Analysis of Randomized Controlled Trials. Circulation.

[5] Kaza N., Keene D., Whinnett Z.I. (2022). Generating Evidence to Support the Physiologic Promise of Conduction System Pacing: Status and Update on Conduction System Pacing Trials. Card. Electrophysiol. Clin..

[6] Daubert C., Behar N., Martins R.P., Mabo P., Leclercq C. (2017). Avoiding non-responders to cardiac resynchronization therapy: A practical guide. Eur. Heart J..

[7] Hejjel L., Németh M., Melczer L., Kónyi A. (2021). Cardiac resynchronization therapy with intraoperative epicardial mapping via minithoracotomy: 10 years’ experience. Pacing Clin. Electrophysiol..

[8] Gold M.R., Leman R.B., Wold N., Sturdivant J.L., Yu Y. (2014). The Effect of Left Ventricular Electrical Delay on the Acute Hemodynamic Response with Cardiac Resynchronization Therapy. J. Cardiovasc. Electrophysiol..

[9] Gold M.R., Birgersdotter-Green U., Singh J.P., Ellenbogen K.A., Yu Y., Meyer T.E., Seth M., Tchou P.J. (2011). The relationship between ventricular electrical delay and left ventricular remodelling with cardiac resynchronization therapy. Eur. Heart J..

[10] Field M.E., Yu N., Wold N., Gold M.R. (2020). Comparison of measures of ventricular delay on cardiac resynchronization therapy response. Heart Rhythm.

[11] Singh J.P., Fan D., Heist E.K., Alabiad C.R., Taub C., Reddy V., Mansour M., Picard M.H., Ruskin J.N., Mela T. (2006). Left ventricular lead electrical delay predicts response to cardiac resynchronization therapy. Heart Rhythm.

[12] Roubicek T., Wichterle D., Kucera P., Nedbal P., Kupec J., Sedlakova J., Cerny J., Stros J., Kautzner J., Polasek R. (2015). Left Ventricular Lead Electrical Delay Is a Predictor of Mortality in Patients With Cardiac Resynchronization Therapy. Circ. Arrhythm. Electrophysiol..

[13] Waddingham P.H., Lambiase P., Muthumala A., Rowland E., Chow A.W. (2021). Fusion Pacing with Biventricular, Left Ventricular-only and Multipoint Pacing in Cardiac Resynchronisation Therapy: Latest Evidence and Strategies for Use. Arrhythmia Electrophysiol. Rev..

[14] Passafaro F., Rapacciuolo A., Ruocco A., Ammirati G., Crispo S., Pasceri E., Santarpia G., Mauro C., Esposito G., Indolfi C. (2024). COMPArison of Multi-Point Pacing and ConvenTional Cardiac Resynchronization Therapy Through Noninvasive Hemodynamics Measurement: Short- and Long-Term Results of the COMPACT-MPP Study. Am. J. Cardiol..

[15] Wang Y., Zhu H., Hou X., Wang Z., Zou F., Qian Z., Wei Y., Wang X., Zhang L., Li X. (2022). Randomized Trial of Left Bundle Branch vs Biventricular Pacing for Cardiac Resynchronization Therapy. J. Am. Coll. Cardiol..

[16] Huang W., Wu S., Vijayaraman P., Su L., Chen X., Cai B., Zou J., Lan R., Fu G., Mao G. (2020). Cardiac Resynchronization Therapy in Patients with Nonischemic Cardiomyopathy Using Left Bundle Branch Pacing. JACC Clin. Electrophysiol..

[17] Li X., Qiu C., Xie R., Ma W., Wang Z., Li H., Wang H., Hua W., Zhang S., Yao Y. (2020). Left bundle branch area pacing delivery of cardiac resynchronization therapy and comparison with biventricular pacing. ESC Heart Fail..

[18] Pujol-Lopez M., Jiménez-Arjona R., Garre P., Guasch E., Borràs R., Doltra A., Ferró E., García-Ribas C., Niebla M., Carro E. (2022). Conduction System Pacing vs Biventricular Pacing in Heart Failure and Wide QRS Patients: LEVEL-AT Trial. JACC Clin. Electrophysiol..

[19] Vijayaraman P., Zalavadia D., Haseeb A., Dye C., Madan N., Skeete J.R., Vipparthy S.C., Young W., Ravi V., Rajakumar C. (2022). Clinical outcomes of conduction system pacing compared to biventricular pacing in patients requiring cardiac resynchronization therapy. Heart Rhythm.

[20] Rijks J., Luermans J., Vernooy K. (2023). Left bundle branch–optimized cardiac resynchronization therapy: Pursuing the optimal resynchronization in severe (distal) conduction system disease. Heart Case Rep..

[21] Vijayaraman P. (2021). Left Bundle Branch Pacing Optimized Cardiac Resynchronization Therapy: A Novel Approach. JACC Clin. Electrophysiol..

[22] Jastrzębski M., Moskal P., Huybrechts W., Curila K., Sreekumar P., Rademakers L.M., Ponnusamy S.S., Herweg B., Sharma P.S., Bednarek A. (2022). Left bundle branch–optimized cardiac resynchronization therapy (LOT-CRT): Results from an international LBBAP collaborative study group. Heart Rhythm.

[23] Chen X., Li X., Bai Y., Wang J., Qin S., Bai J., Wang W., Liang Y., Chen H., Su Y. (2023). Electrical Resynchronization and Clinical Outcomes During Long-Term Follow-Up in Intraventricular Conduction Delay Patients Applied Left Bundle Branch Pacing-Optimized Cardiac Resynchronization Therapy. Circ. Arrhythm. Electrophysiol..

[24] Burri H., Starck C., Auricchio A., Biffi M., Burri M., D’Avila A., Deharo J.-C., Glikson M., Israel C., Lau C.-P. (2021). EHRA expert consensus statement and practical guide on optimal implantation technique for conventional pacemakers and implantable cardioverter-defibrillators: Endorsed by the Heart Rhythm Society (HRS), the Asia Pacific Heart Rhythm Society (APHRS), and the Latin-American Heart Rhythm Society (LAHRS). EP Eur..

[25] Singh J.P., Klein H.U., Huang D.T., Reek S., Kuniss M., Quesada A., Barsheshet A., Cannom D., Goldenberg I., McNitt S. (2011). Left Ventricular Lead Position and Clinical Outcome in the Multicenter Automatic Defibrillator Implantation Trial–Cardiac Resynchronization Therapy (MADIT-CRT) Trial. Circulation.

[26] Burri H., Jastrzebski M., Cano Ó., Čurila K., de Pooter J., Huang W., Israel C., Joza J., Romero J., Vernooy K. (2023). EHRA clinical consensus statement on conduction system pacing implantation: Endorsed by the Asia Pacific Heart Rhythm Society (APHRS), Canadian Heart Rhythm Society (CHRS), and Latin American Heart Rhythm Society (LAHRS). EP Eur..

[27] Ezer P., Farkas N., Szokodi I., Kónyi A. (2021). Automatic daily remote monitoring in heart failure patients implanted with a cardiac resynchronisation therapy-defibrillator: A single-centre observational pilot study. Arch. Med. Sci. AMS.

[28] Singh J.P., Berger R.D., Doshi R.N., Lloyd M., Moore D., Stone J., Daoud E.G. (2020). Null, null Targeted Left Ventricular Lead Implantation Strategy for Non-Left Bundle Branch Block Patients. JACC Clin. Electrophysiol..

[29] Cleland J.G., Abraham W.T., Linde C., Gold M.R., Young J.B., Claude Daubert J., Sherfesee L., Wells G.A., Tang A.S.L. (2013). An individual patient meta-analysis of five randomized trials assessing the effects of cardiac resynchronization therapy on morbidity and mortality in patients with symptomatic heart failure. Eur. Heart J..

[30] Jastrzębski M., Baranchuk A., Fijorek K., Kisiel R., Kukla P., Sondej T., Czarnecka D. (2019). Cardiac resynchronization therapy-induced acute shortening of QRS duration predicts long-term mortality only in patients with left bundle branch block. EP Eur..

[31] Huang W., Su L., Wu S., Xu L., Xiao F., Zhou X., Ellenbogen K.A. (2017). A Novel Pacing Strategy with Low and Stable Output: Pacing the Left Bundle Branch Immediately Beyond the Conduction Block. Can. J. Cardiol..

[32] Su L., Wang S., Wu S., Xu L., Huang Z., Chen X., Zheng R., Jiang L., Ellenbogen K.A., Whinnett Z.I. (2021). Long-Term Safety and Feasibility of Left Bundle Branch Pacing in a Large Single-Center Study. Circ. Arrhythm. Electrophysiol..

[33] Zhang W., Huang J., Qi Y., Wang F., Guo L., Shi X., Wu W., Zhou X., Li R. (2019). Cardiac resynchronization therapy by left bundle branch area pacing in patients with heart failure and left bundle branch block. Heart Rhythm.

[34] Diaz J.C., Sauer W.H., Duque M., Koplan B.A., Braunstein E.D., Marín J.E., Aristizabal J., Niño C.D., Bastidas O., Martinez J.M. (2023). Left Bundle Branch Area Pacing Versus Biventricular Pacing as Initial Strategy for Cardiac Resynchronization. JACC Clin. Electrophysiol..

